# Supplementary material for: Social determinants of tobacco use among tribal communities in India: Evidence from the first wave of Longitudinal Ageing Study in India
Source: PLoS One. 2023 Mar 2;18(3):e0282487. doi: 10.1371/journal.pone.0282487 (PMC9980830; doi:10.1371/journal.pone.0282487)
Supplement: S3 Table — (DOCX) [file pone.0282487.s003.docx]

**Supplementary table 3. State wise prevalence of different forms of tobacco in India**

| **State** | **Smoking** | **SLT** | **Any tobacco** |
| --- | --- | --- | --- |
| Andaman & Nicobar Island | 13.48 | 30.95 | 39.63 |
| Andhra Pradesh | 25.74 | 7.66 | 33.4 |
| Arunachal Pradesh | 11.98 | 9.01 | 17.45 |
| Assam | 10.53 | 42.05 | 46.53 |
| Bihar | 31.34 | 36.13 | 59.3 |
| Chandigarh | 30.17 | 2.64 | 30.17 |
| Chhattisgarh | 13.62 | 36.82 | 48.62 |
| Dadra Nagar | 15.63 | 27.31 | 39.36 |
| Daman and Diu | 12.66 | 22.14 | 31.33 |
| Goa | 8.1 | 22.05 | 30.16 |
| Gujarat | 12.22 | 30.69 | 41.8 |
| Haryana | 30.93 | 12.81 | 30.93 |
| Himachal Pradesh | 30.9 | 2.68 | 30.9 |
| Jammu & Kashmir | 17.63 | 16.01 | 33.23 |
| Jharkhand | 5.94 | 44.82 | 46.72 |
| Karnataka | 18.82 | 19.08 | 34.49 |
| Kerala | 37.07 | 11.57 | 40.87 |
| Ladakh | 0 | 0 | 0 |
| Lakshadweep | 9.24 | 21.82 | 28.77 |
| Madhya Pradesh | 19.84 | 22.4 | 38.78 |
| Maharashtra | 10.78 | 35.42 | 43.45 |
| Manipur | 34.99 | 34.72 | 55.71 |
| Meghalaya | 22.33 | 16.55 | 37.44 |
| Mizoram | 46.71 | 43.37 | 78.69 |
| Nagaland | 16.24 | 16.64 | 25.72 |
| Delhi | 8.81 | 0 | 8.81 |
| Odisha | 12.25 | 57.91 | 60.82 |
| Puducherry | 23.71 | 0 | 23.7 |
| Punjab | 0.64 | 0 | 0.64 |
| Rajasthan | 39.57 | 8.66 | 46.29 |
| Sikkim | 0 | 0 | 0 |
| Tamil Nadu | 9.63 | 11.19 | 20.82 |
| Telangana | 36.09 | 17.24 | 52.38 |
| Tripura | 50.47 | 39.93 | 78.72 |
| Uttar Pradesh | 17.15 | 48.4 | 61.31 |
| Uttarakhand | 24.24 | 10.55 | 33.36 |
| West Bengal | 23.19 | 35.01 | 49.99 |
