# Supplementary material for: Social determinants of tobacco use among tribal communities in India: Evidence from the first wave of Longitudinal Ageing Study in India
Source: PLoS One. 2023 Mar 2;18(3):e0282487. doi: 10.1371/journal.pone.0282487 (PMC9980830; doi:10.1371/journal.pone.0282487)
Supplement: Attachment — Submitted filename: PONE-D-22-13582.pdf [file pone.0282487.s004.pdf]

PLOS ONE

Social determinants of tobacco use among tribal communities in India: evidence on ethnicity and tobacco use from LASI, wave-1

--Manuscript Draft--

**Manuscript Number:**	PONE-D-22-13582
**Article Type:**	Research Article
**Full Title:**	Social determinants of tobacco use among tribal communities in India: evidence on ethnicity and tobacco use from LASI, wave-1
**Short Title:**	Ethnicity and tobacco use
**Corresponding Author:**	Sanghamitra Pati, M.D., MPH<br>Regional Medical Research Centre Bhubaneswar<br>Bhubaneswar, Odisha INDIA
**Keywords:**	tobacco; LASI,; tribal; ethnicity; older adults; smoking; smokeless tobacco
**Abstract:**	ABSTRACT<br>Background<br>Evidence on tobacco use among indigenous communities is scarce; mainly based on either specific region or from particular tribe. Considering the large tribal population in India, it is pertinent to generate evidence on tobacco use among this community. We aimed to estimate the prevalence of tobacco use, assess its determinants and regional variations among older tribal adults in India using nationally representative data.<br>Methods<br>We analysed data from Longitudinal Ageing Study in India (LASI) wave-1 conducted in 2017-18. 11,365 tribal individuals aged ≥ 45 years were included in this study. Descriptive statistics was used to assess the socio-demographic profiles of the participants, expressed as frequency and proportions. Separate multivariable regression models were executed to find the association of various socio-demographic variables with different forms of tobacco, reported as adjusted odds ratio with 95% confidence interval.<br>Findings<br>The overall prevalence of tobacco use was found to be around 46%; with 19% of them smokers and nearly 32% of SLT (Smokeless tobacco) users. Participants from the most deprived group had a significantly higher risk of consuming smokeless tobacco users [Adjusted odds ratio (AOR) : 1.38 (1.01-1.87]. Alcohol was found to be associated with both smoking [AOR: 2.09 (1.69-2.57)] and smokeless [AOR: 3.05 (2.54-3.66)]. Relatively higher odds of smoking was evident from the central region [AOR: 2.75 (1.97-3.84)] while smokeless tobacco was associated with the eastern region [AOR: 6.21 (3.91-9.88)].<br>Interpretation<br>This study highlights a high burden of tobacco use along with its social determinants which can help in tailoring anti-tobacco messages for target population to make tobacco control programs more effective.
**Order of Authors:**	Jogesh Murmu, MPH
	Ritik Agrawal, BDS, MPH
	Sayantani Manna, MPH
	Sweta Pattnaik, MPH
	Shishirendu Ghosal, BDS, MPH
	Abhinav Sinha, BDS, MPH
	Ardhendu Sekhar Acharya, PhD
	Srikanta Kanungo, MBBS, MD
	Sanghamitra Pati, MBBS, MD, MPH
**Additional Information:**	

Powered by Editorial Manager® and ProduXion Manager® from Aries Systems Corporation

Question	Response
**Financial Disclosure**<br><br>Enter a financial disclosure statement that describes the sources of funding for the work included in this submission. Review the submission guidelines for detailed requirements. View published research articles from *PLOS ONE* for specific examples.<br><br>This statement is required for submission and **will appear in the published article** if the submission is accepted. Please make sure it is accurate.<br><br>**Unfunded studies**<br>Enter: *The author(s) received no specific funding for this work.*<br><br>**Funded studies**<br>Enter a statement with the following details:<br>• Initials of the authors who received each award<br>• Grant numbers awarded to each author<br>• The full name of each funder<br>• URL of each funder website<br>• Did the sponsors or funders play any role in the study design, data collection and analysis, decision to publish, or preparation of the manuscript?<br>• **NO** - Include this sentence at the end of your statement: *The funders had no role in study design, data collection and analysis, decision to publish, or preparation of the manuscript.*<br>• **YES** - Specify the role(s) played.<br><br>* typeset	The author(s) received no specific funding for this work.
**Competing Interests**<br><br>Use the instructions below to enter a competing interest statement for this submission. On behalf of all authors, disclose any competing interests that could be perceived to bias this work—acknowledging all financial support and any other relevant financial or non-financial competing interests.	The authors have declared that no competing interests exist.

This statement is required for submission and **will appear in the published article** if the submission is accepted. Please make sure it is accurate and that any funding sources listed in your Funding Information later in the submission form are also declared in your Financial Disclosure statement.<br><br>View published research articles from *PLOS ONE* for specific examples.<br><br>**NO authors have competing interests**<br><br>Enter: *The authors have declared that no competing interests exist.*<br><br>**Authors with competing interests**<br><br>Enter competing interest details beginning with this statement:<br><br>*I have read the journal's policy and the authors of this manuscript have the following competing interests: [insert competing interests here]*<br><br>* typeset	
**Ethics Statement**<br><br>Enter an ethics statement for this submission. This statement is required if the study involved:<br><br>• Human participants<br>• Human specimens or tissue<br>• Vertebrate animals or cephalopods<br>• Vertebrate embryos or tissues<br>• Field research<br><br>Write "N/A" if the submission does not require an ethics statement.<br><br>General guidance is provided below. Consult the submission guidelines for detailed instructions. **Make sure that all information entered here is included in the Methods section of the manuscript.**	LASI received mandatory ethical approval from the Indian Council of Medical Research and Institutional Review Board (IRB) held at IIPS, Mumbai. Written consent forms were administered at household and individual levels, following the Human Subject Protection. LASI data is archived in a public repository; therefore, there is no need for additional ethical approval to conduct the present study.

Powered by Editorial Manager® and ProduXion Manager® from Aries Systems Corporation

**Format for specific study types**<br><br>**Human Subject Research (involving human participants and/or tissue)**<br>• Give the name of the institutional review board or ethics committee that approved the study<br>• Include the approval number and/or a statement indicating approval of this research<br>• Indicate the form of consent obtained (written/oral) or the reason that consent was not obtained (e.g. the data were analyzed anonymously)<br><br>**Animal Research (involving vertebrate animals, embryos or tissues)**<br>• Provide the name of the Institutional Animal Care and Use Committee (IACUC) or other relevant ethics board that reviewed the study protocol, and indicate whether they approved this research or granted a formal waiver of ethical approval<br>• Include an approval number if one was obtained<br>• If the study involved *non-human primates*, add *additional details* about animal welfare and steps taken to ameliorate suffering<br>• If anesthesia, euthanasia, or any kind of animal sacrifice is part of the study, include briefly which substances and/or methods were applied<br><br>**Field Research**<br><br>Include the following details if this study involves the collection of plant, animal, or other materials from a natural setting:<br>• Field permit number<br>• Name of the institution or relevant body that granted permission	
**Data Availability**<br><br>Authors are required to make all data underlying the findings described fully available, without restriction, and from the time of publication. PLOS allows rare exceptions to address legal and ethical concerns. See the PLOS Data Policy and FAQ for detailed information.	Yes - all data are fully available without restriction

Powered by Editorial Manager® and ProduXion Manager® from Aries Systems Corporation

A Data Availability Statement describing where the data can be found is required at submission. Your answers to this question constitute the Data Availability Statement and **will be published in the article**, if accepted.<br><br>**Important:** Stating 'data available on request from the author' is not sufficient. If your data are only available upon request, select 'No' for the first question and explain your exceptional situation in the text box.<br><br>Do the authors confirm that all data underlying the findings described in their manuscript are fully available without restriction?	
**Describe where the data may be found in full sentences. If you are copying our sample text, replace any instances of XXX with the appropriate details.**<br><br>• If the data are **held or will be held in a public repository**, include URLs, accession numbers or DOIs. If this information will only be available after acceptance, indicate this by ticking the box below. For example: *All XXX files are available from the XXX database (accession number(s) XXX, XXX.*).<br>• If the data are all contained **within the manuscript and/or Supporting Information files**, enter the following: *All relevant data are within the manuscript and its Supporting Information files.*<br>• If neither of these applies but you are able to provide **details of access elsewhere**, with or without limitations, please do so. For example:<br><br>*Data cannot be shared publicly because of [XXX]. Data are available from the XXX Institutional Data Access / Ethics Committee (contact via XXX) for researchers who meet the criteria for access to confidential data.*<br><br>*The data underlying the results presented in the study are available*	The dataset analysed during the current study is available in the LASI data repository held at ICT, IIPS [https://g2aging.org/?section=overviews&study=lasi].

from (include the name of the third party and contact information or URL).<br>• This text is appropriate if the data are owned by a third party and authors do not have permission to share the data.<br><br>* typeset	
Additional data availability information:	

Powered by Editorial Manager® and ProduXion Manager® from Aries Systems Corporation

Manuscript

Click here to access/download;Manuscript;LASI_final_manuscript_NEW_v3.

Social determinants of tobacco use among tribal communities in India: evidence on ethnicity and tobacco use from LASI, wave-1

Jogesh Murmu[1#], Ritik Agrawal[1#], Sayantani Manna[1#], Sweta Pattnaik[1#], Shishirendu Ghosal[1#], Abhinav Sinha[2], Ardhendu Sekhar Acharya[1], Srikanta Kanungo[1*], Sanghamitra Pati[1*]

[#]Authors with equal contribution

Mr Jogesh Murmu
ICMR-Regional Medical Research Centre
Bhubaneswar, Odisha, India
Email: mjogesh14@gmail.com
ORCID ID: https://orcid.org/0000-0001-9428-9113

Dr Ritik Agrawal
ICMR-Regional Medical Research Centre
Bhubaneswar, Odisha, India
Email: 0909ritik@gmail.com
ORCID ID: https://orcid.org/0000-0001-5345-1559

Ms. Sayantani Manna
ICMR-Regional Medical Research Centre
Bhubaneswar, Odisha, India
Email: sayantanimanna6@gmail.com
ORCID ID: https://orcid.org/0000-0001-9093-1172

Ms. Sweta Pattnaik
ICMR-Regional Medical Research Centre
Bhubaneswar, Odisha, India
Email: sweta.pattnaik132@gmail.com
ORCID ID: https://orcid.org/0000-0002-6888-9980

Dr Shishirendu Ghosal
ICMR-Regional Medical Research Centre
Bhubaneswar, Odisha, India
Email: shishirendu123@gmail.com
ORCID ID: https://orcid.org/0000-0003-1833-3703

Dr Abhinav Sinha
Health Technology Assessment in India (HTAIn),
Department of Health Research
ICMR-Regional Medical Research Centre
Bhubaneswar, Odisha, India
Email: dr.abhinav17@gmail.com
ORCID ID: https://orcid.org/0000-0001-7702-3671

Dr Ardhendu Sekhar Acharya
ICMR-Regional Medical Research Centre
Bhubaneswar, Odisha, India
Email: asarmrc@gmail.com

Dr Srikanta Kanungo
ICMR-Regional Medical Research Centre
Bhubaneswar, Odisha, India
Email: srikantak109@gmail.com
ORCID ID: https://orcid.org/0000-0001-5647-0122

Dr Sanghamitra Pati
ICMR-Regional Medical Research Centre
Bhubaneswar, Odisha, India
Email: drsanghamitra12@gmail.com
ORCID ID: https://orcid.org/0000-0002-7717-5592

***Correspondence to:**

Dr Sanghamitra Pati, drsanghamitra12@gmail.com

Dr Srikanta Kanungo, srikantak109@gmail.com

ABSTRACT

Background

Evidence on tobacco use among indigenous communities is scarce; mainly based on either specific region or from particular tribe. Considering the large tribal population in India, it is pertinent to generate evidence on tobacco use among this community. We aimed to estimate the prevalence of tobacco use, assess its determinants and regional variations among older tribal adults in India using nationally representative data.

Methods

We analysed data from Longitudinal Ageing Study in India (LASI) wave-1 conducted in 2017-18. 11,365 tribal individuals aged ≥ 45 years were included in this study. Descriptive statistics was used to assess the socio-demographic profiles of the participants, expressed as frequency and proportions. Separate multivariable regression models were executed to find the association of various socio-demographic variables with different forms of tobacco, reported as adjusted odds ratio with 95% confidence interval.

Findings

The overall prevalence of tobacco use was found to be around 46%; with 19% of them smokers and nearly 32% of SLT (Smokeless tobacco) users. Participants from the most deprived group had a significantly higher risk of consuming smokeless tobacco users [Adjusted odds ratio (AOR) : 1.38 (1.01-1.87]. Alcohol was found to be associated with both smoking [AOR: 2.09 (1.69-2.57)] and smokeless [AOR: 3.05 (2.54-3.66)]. Relatively higher odds of smoking was evident from the central region [AOR: 2.75 (1.97-3.84)] while smokeless tobacco was associated with the eastern region [AOR: 6.21 (3.91-9.88)].

Interpretation

This study highlights a high burden of tobacco use along with its social determinants which can help in tailoring anti-tobacco messages for target population to make tobacco control programs more effective.

Funding: No funds were received to conduct the present research.

Key words: tobacco, LASI, tribal, ethnicity, older adults, smoking, smokeless tobacco

INTRODUCTION

Globally, tobacco use is one of the greatest public health threats. The rampant use of various tobacco products is a matter of concern in low-and middle-income countries (LMICs). Evidence suggests that almost 1.3 billion people use tobacco in the world, out of which 80% live in LMICs, where the prevalence of tobacco-related morbidities and mortality is highest.(1) Among LMICs, India is the second-largest consumer and still a large-scale producer of tobacco products.2, (3) The tobacco ecosystem in India is complex.(4) According to Global Adult Tobacco Survey-2 (GATS-2, 2016-17), there were nearly 267 million tobacco users aged ≥15 years in India and among them about 42.4% were men and 14.2% were women.(5)

Tobacco is commonly used in three ways i.e., smokeless, smoking and dual form. Due to socio-cultural acceptability, smokeless tobacco is highly prevalent in India which includes chewing tobacco such as khaini, gutkha, betel quid with tobacco, mishri, gul, and gudakhu.(6) Other kinds include smoking (cigarette, hookah, and bidi), and use of any form of tobacco which simultaneously predispose a risk of oral submucous fibrosis (OSMF), a premalignant disorder with a potency to transform into oral cancer.(7) Evidence supports that smoking is responsible for health ailments of the cardiovascular and respiratory systems.(6)

Tobacco not only results in loss of lives but also levies associated social and economic costs. In India, the total economic cost of tobacco use for all illnesses during 2017-18 among people aged ≥35 years is approximately INR 177 billion (US $ 27.5 billion).(8)

India is a diversified land of many cultures, ethnic groups with one of the largest tribal populations in the world. According to the Census of India-2011, the scheduled tribes (STs) comprised of around 104 million people which is 8.6% of the national population.(9) There are 550 different tribes in India including particularly vulnerable tribal groups (PVTGs). Approximately 90% of the country’s tribal population live in rural areas, while the remaining

10% reside in urban areas.(10) Still, a gap exists between the indigenous and non-tribal populations as the former prefer to live in their own geographical habitats which are often secluded in forests and hard-to-reach areas. Their culture and traditions largely vary which disintegrates them from mainstream socio-economic activities; a life often marred with subsistence-based existence, invariably leading to their lower education (41% with no formal education) and socio-economic attainment (41% below poverty line).(11) This social exclusion often compels these marginalized communities to disparities in accessing public health services.

Moreover, deleterious habits such as tobacco use is deeply rooted in their social beliefs and cultural practices. It often remains entrenched in their practices which is difficult to mould. As per the social affirmation and traditional practices, the pervasiveness of tobacco consumption is quite obvious. For instance, SLT is wrongly perceived as safer than smoking which ends up in higher consumption, early initiation, and persistence as a norm.(12,13) Among the indigenous communities mortality, morbidity, and malnutrition rates are still larger as compared to the average Indian population which is a result of various barriers such as language, education, and infrastructural advancements.(14)

The habitual consumption of tobacco use has been an eminent practice among tribal communities since ancient times.(15) Tobacco use among the general population is widely studied based on the data available from Global Youth Tobacco Survey (GYTS) and Global Adult Tobacco Survey (GATS), but it is scarce in context of tribal population.(16,17) Most of the evidence on tobacco use among tribe is either from specific region or based on survey from particular tribe with no nationally representative study. Considering the large tribal population in India and their unique health-related behaviours, it is pertinent to generate evidence on tobacco use among this community so as to pave a way for future tobacco control programs

and policies targeting this marginalized group. Hence, we aimed to estimate the prevalence of tobacco use, assess its determinants and regional variations among older tribal adults in India using nationally representative data.

MATERIALS AND METHODS

Overview of data

This study is based on the Longitudinal Aging Study of India (LASI), wave-1, which was a full-scale national survey to scientifically investigate health, economics, social determinants, and consequences of population ageing in India; conducted during April 2017 to December 2018, by Harvard TH Chan School of Public Health, the University of Southern California in partnership with International Institute for Population Sciences (IIPS), Mumbai. LASI used three instruments for data collection viz., household survey schedule, individual survey schedule and community survey schedule. LASI interviewed a sample of 72,250 individuals aged ≥45 years (and their spouses irrespective of age) who provided written consent before the interviews. A multistage stratified area probability cluster sampling design was employed to reach the final unit of observation.(18) The detailed method for LASI, wave-1 has been mentioned on the website of IIPS, Mumbai.(19) We followed Strengthening the Reporting of Observational studies in Epidemiology (STROBE) guideline to report this study (Supplementary file S1).(20)

Study participants and sample size

An individual survey schedule was employed among 72,250 participants aged ≥45 years. Among them, participants who referred their caste to be "Scheduled tribes" (STs) were included in this study. Following this, the conclusive sample size of 11,365 tribal individuals aged ≥45 years was achieved as per the objective of this study.

<Figure 1: Selection of study population>

Independent Variables/Covariates

We employed various socio-demographic variables such as age, gender (male/female), residence (rural/urban), education, occupation, partner status, regions of the country, and wealth index. Additionally, we included two attributes on personal behavioural i.e., physical activity and alcohol consumption in the analysis. Age was categorized into three groups – 45 to 59 years, 60 to 74 years, and ≥75 years. Education was stratified into four categories as "no formal education", "up to standard VII", "standard VIII-X" and "higher secondary and above". Respondents who did not work more than three months in their lifetime and were not employed during the survey were grouped as "currently not working" while the rest were "currently working". Couples who were married or lived together (live-in) were grouped into "living with partner" while the rest (widowed, divorced, separated, deserted and never married) were clubbed into "living without partner". 29 States and 6 Union Territories (except Sikkim) of India were arranged into six regions (north, central, east, north-east, west, and south) based on their geographical location. Participants who were hardly or never involved in sports or vigorous activities were grouped as "physically inactive", while others (daily to as less frequent as "one to three times a month") were merged into "physically active" group.

Outcome Variable

The outcome variable for this analysis was tobacco use i.e., smoking, smokeless, and any form of tobacco use among the tribal population in India. Status of tobacco use was classified based on the combined responses from the following two questions, first: "Have you ever smoked or used smokeless tobacco?", those who responded 'yes' were further enquired: "what type of tobacco product have you used or consumed?" with options as smoking, smokeless and both smoking and smokeless tobacco. Smokers, as well as users of tobacco in dual form (smoking

and smokeless), were clubbed as “smoking”. Next, the users of smokeless tobacco and dual users were grouped as “smokeless tobacco” users. Additionally, a third group of “any tobacco” users was formed by clubbing participants who smoked or used smokeless tobacco or consumed the dual form of tobacco.

Statistical Analysis

Data was analyzed using STATA, v16·0 (STATA Corp., Texas) for Windows. Descriptive analysis was presented for continuous variables, such as age in mean with standard deviation (SD). The frequency and proportions (n, %) of tobacco use for each sub-group were described for categorical variables. The distribution of tobacco consumption in all three forms was tabulated with socio-demographic and behavioral covariates. 95% confidence interval (CI) for all weighted proportions was reported as a measure of uncertainty. We considered p-value of <0.05 to be significant. Univariate logistic regression was applied to determine the crude odds ratio between distinct outcomes and attributes. Significantly associated variables were used in separate multivariable models to obtain adjusted association between outcome variables and various correlates, presented as adjusted odds ratio with 95% CI. Sampling weights were considered in the analysis to adjust for multistage sampling design.

Ethical consideration

Indian Council of Medical Research (ICMR), New Delhi and IIPS, Mumbai granted ethical approval for the first wave of the LASI survey. However, we used secondary anonymous data which is available in the public domain, hence there is no participant risk. Appropriate permission to use the dataset was received and data is being acknowledged wherever entailed.

RESULTS

For this analysis, eventually, 11,365 schedule tribes aged 45 years and above, were included. Their average age was 59.3 (±10·7) years. The majority of them belonged to the age group of 45-59 years (54.5%). Nearly equal number of male and female interviewees were included with a little female predilection (53.7%). A greater part of the tribal population never went to school (54.6%) and lived in a rural setup (77.4%). Most of the participants were physically inactive (52.6%) and worked (56.2%) while interviewed (Table 1).

Table 1: Sociodemographic characteristics of study population (n=11,365)

**Socio-demographic Characteristics**		**n (%)**
Age	45 to 59 years	6192 (54.5%)
	60 to 74 years	4058 (35.7%)
	≥75 years	1115 (9.8%)
Gender	Male	5256 (46.3%)
	Female	6109 (53.7%)
Residence	Rural	8801 (77.4%)
	Urban	2564 (22.6%)
Education	No formal education	6205 (54.6%)
	Up to standard VII	2942 (25.9%)
	Standard VIII-X	1633 (14.4%)
	Higher secondary & above	584 (5.1%)
Occupation	Currently working	6383 (56.2%)
	Currently not working	4977 (43.8%)
Life-partner	Living with partner	8349 (73.5%)
	Living without partner	3016 (26.5%)
Region	North	367 (3.2%)
	Central	1440 (12.7%)
	East	1257 (11.1%)
	North East	4951 (43.6%)
	West	1663 (14.6%)
	South	1687 (14.8%)
Physical Activity	Active	5346 (47.4%)
	Not Active	5941 (52.6%)
Alcohol	Yes	3267 (28.9%)
	No	8021 (71.1%)
Asset Quintile	Most deprived	3274 (28.8%)
	2	2438 (21.5%)
	3	2066 (18.2%)
	4	1776 (15.6%)
	Most affluent	1811 (15.9%)

The overall prevalence of tobacco use was 46.1%, while smoking and smokeless tobacco contributed to 18.6% and 31.7% respectively; tobacco in both forms (dual use) was consumed by 4.2% of the participants. Smoking and smokeless tobacco were more prevalent among males (smoking: 34.8% and smokeless tobacco: 40.1%) (Figure 2).

<Figure-2: Type of tobacco consumption across gender among the tribal populations>

The habit of smoking and smokeless tobacco use was highest (23.7% and 38.4% respectively) among 75 years old and above. Tribals from southern India were most likely to smoke (24.1%), while the eastern tribal population mostly (48.6%) used smokeless tobacco. State-wise distribution of tobacco use in smoking or smokeless form along with any tobacco use has been presented in (Figure 3a,3b,3c). The detailed state wise prevalence of various forms of tobacco is presented in supplementary table -2.

< **Figure 3a: State wise prevalence of smoking tobacco among tribal population in India**>

< Figure 3b: State wise prevalence of smokeless tobacco among tribal population in India>

< Figure 3c: State wise prevalence of any tobacco among tribal population in India>

Tobacco use of any variant was relatively higher among alcohol consumers (32.9% smokers, 52.5% smokeless tobacco, and 75.8% used any kind of tobacco). The physically active participants used tobacco of any form more (smokers: 20.0%, smokeless tobacco: 36.8%) than those who were not into sports or heavy physical work regularly (smokers: 17.3% and smokeless tobacco: 26.3%). The detailed distribution of tobacco use is provided in Table 2.

Table-2 Prevalence of various forms of tobacco and its univariate association with socio-demographic characteristics.

Socio-demographic Characteristics	Smoking		Smokeless Tobacco		Any Tobacco	
	**n (%)**	**OR, 95% CI**	**n (%)**	**OR, 95% CI**	**n (%)**	**OR, 95% CI**
Age						
45 to 59 years	1014, 17.0% (16.0-18.0)	Reference	1861, 31.2% (30.0-32.4)	1.02 (0.86-1.22)	2640, 44.2% (42.9-45.5)	Reference
60 to 74 years	858, 19.6% (18.5-20.9)	1.19 (0.97-1.47)	1342, 30.7% (29.4-32.1)	Reference	2039, 46·7% (45·2-48·2)	1·10 (0·93-1·31)
≥75 years	243, 23.7% (21.1-26.4)	1.52 (1.06-2.19)	393, 38.4% (35.4-41.4)	1.41 (1.01-1.95)	557, 54·4% (51·3-57·5)	1·50 (1·13-2·01)
Gender						
Male	1790, 34·8% (33·5-36·1)	9·67 (7·84-11·92)	2063, 40·1% (38·7-41·4)	2·04 (1·73-2·40)	3417, 66·4% (65·1-67·7)	4·77 (4·06-5·61)
Female	325 (5·2%) (4·7-5·8)	Reference	1533, 24·7% (23·6-25·8)	Reference	1819, 29·3% (28·1-30·4)	Reference
Residence						
Rural	1952, 19·5% (18·7-20·3)	1·77 (1·21-2·59)	3240, 32·4% (31·4-33·3)	1·34 (1·00-1·79)	4734, 47·3% (46·3-48·3)	1·52 (1·14-2·04)
Urban	163, 12·0% (10·4-13·9)	Reference	356, 26·3% (24·0-28·7)	Reference	502, 37·1% (34·5-39·7)	Reference
Education						
No formal education	1313, 17·2% (16·4-18·1)	1·08 (0·78-1·48)	2306, 30·3% (29·2-31·3)	1·56 (1·02-2·38)	3389, 44·5% (43·3-45·6)	1·56 (1·05-2·32)
Up to standard VII	556, 24·8% (23·0-26·7)	1·71 (1·20-2·44)	840, 37·5% (35·5-39·5)	2·15 (1·37-3·38)	1227, 54·8% (52·7-56·9)	2·36 (1·54-3·62)
Standard VIII-X	172, 16·2% (14·1-18·6)	Reference	354, 33·4% (30·6-36·3)	1·80 (1·11-2·92)	470, 44·3% (41·3-47·4)	1·55 (0·98-2·45)
Higher secondary & above	74, 16·8% (13·4-20·6)	1·05 (0·62-1·77)	96, 21·8% (18·0-25·9)	Reference	150, 33·9% (29·5-38·6)	Reference
Occupation						
Currently working	1412, 20·6% (19·7-21·6)	1·41 (1·13-1·75)	2391, 34·9% (33·8-36·1)	1·47 (1·23-1·76)	3503, 51·2% (50·0-52·4)	1·67 (1·41-1·99)
Currently not working	704, 15·6% (14·6-16·7)	Reference	1208, 26·8% (25·5-28·1)	Reference	1736, 38·5% (37·0-39·9)	Reference
Life-partner						
Living with partner	1771, 21·4% (20·5-22·3)	2·18 (1·74-2·72)	2609, 31·5% (30·5-32·5)	Reference	3974, 48·0% (46·9-49·1)	1·34 (1·12-1·60)
Living without partner	344, 11·1% (10·0-12·3)	Reference	987, 31·9% (30·3-33·6)	1·02 (0·85-1·23)	1262, 40·8% (39·1-42·6)	Reference

Region						
North	37, 19·1% (13·8-25·3)	1·82 (1·16-2·86)	17, 8·5% (5·2-13·7)	Reference	51, 26·3% (20·2-33·1)	Reference
Central	799, 23·7% (22·3-25·2)	2·39 (1·70-3·36)	803, 23·8% (22·4-25·3)	3·35 (2·13-5·26)	1513, 44·9% (43·2-46·6)	2·28 (1·61-3·23)
East	433, 14·9% (13·7-16·3)	1·35 (0·96-1·90)	1407, 48·6% (46·8-50·4)	10·12 (6·55-15·64)	1611, 55·6% (53·8-57·5)	3·51 (2·51-4·92)
North East	307, 22·0% (19·8-24·2)	2·16 (1·60-2·92)	420, 30·1% (27·7-32·5)	4·60 (3·01-7·04)	634, 45·4% (42·7-48·0)	2·33 (1·69-3·21)
West	281, 11·5% (10·3-12·8)	Reference	802, 32·8% (30·1-34·7)	5·24 (3·33-8·23)	1037, 42·5% (40·5-44·5)	2·07 (1·45-2·94)
South	257, 24·1% (21·6-26·8)	2·44 (1·63-3·67)	147, 13·8% (11·8-16·0)	1·72 (1·01-2·93)	391, 36·6% (33·8-39·7)	1·62 (1·06-2·48)
Physical Activity						
Active	1206, 20·0% (19·0-21·0)	1·19 (0·98-1·46)	2222, 36·8% (35·6-38·1)	1·63 (1·37-1·94)	3145, 52·2% (50·9-53·4)	1·64 (1·39-1·93)
Not Active	909, 17·3% (16·3-18·3)	Reference	1383, 26·3% (25·1-27·5)	Reference	2099, 39·9% (38·6-41·3)	Reference
Alcohol						
Yes	1198, 32·9% (31·5-34·5)	3·60 (2·96-4·37)	1909, 52·5% (50·9-54·2)	3·90 (3·29-4·61)	2753, 75·8% (74·4-77·2)	6·48 (5·46-7·71)
No	921, 12·0% (11·3-12·8)	Reference	1695, 22·1% (21·2-23·1)	Reference	2494, 32·6% (31·5-33·6)	Reference
Asset Quintile						
Most deprived	619, 16·2% (15·1-17·5)	1·02 (0·80-1·30)	1454, 38·2% (36·6-39·7)	2·01 (1·47-2·74)	1888, 49·5% (47·9-51·1)	1·46 (1·07-1·98)
2	455, 16·0% (14·7-17·4)	Reference	899, 31·6% (29·9-33·4)	1·51 (1·08-2·09)	1276, 44·9% (43·0-46·7)	1·21 (0·88-1·67)
3	421, 21·8% (20·0-25·2)	1·46 (1·12-1·91)	570, 29·5% (27·5-31·6)	1·36 (0·97-1·91)	917, 47·5% (45·2-49·7)	1·34 (0·97-1·86)
4	351, 23·0% (20·9-23·7)	1·57 (1·09-2·25)	379, 24·8% (22·7-27·1)	1·08 (0·72-1·62)	651, 42·6% (40·1-45·2)	1·10 (0·75-1·61)
Most affluent	270, 21·6% (19·3-24·0)	1·45 (1·03-2·03)	294, 23·5% (21·2-25·9)	Reference	504, 40·3% (37·5-43·0)	Reference

Univariate logistic regression models identified participants aged 75 years and above, males, rural residents, lesser years of schooling, currently working, having partner and alcohol were associated with tobacco use (Table 2). Table 3 shows the association of socio-demographic, and behavioral factors with different forms of tobacco products among the study population, after adjusting for all the covariates. There was a 74% greater likelihood of any tobacco use

among the 75+ age group (AOR: 1.74 (1.24-2.43). Males were highly associated with using all forms of tobacco (smoking, smokeless or any form) as compared to the females. Relatively higher odds of smoking was evident from central [AOR: 2.75 (1.97-3.84)] and southern [AOR: 2.31 (1.54-3.46)] tribal population, however, smokeless tobacco was highly associated with eastern [AOR: 6.21 (3.91-9.88)] and western region [AOR: 4.01 (2.49-6.44)]. Alcohol was found to be an attributing factor for any form of tobacco i.e. smoking [AOR: 2.09 (1.69-2.57)] or smokeless [AOR: 3.05 (2.54-3.66)]. Physical activity was associated with SLT [AOR: 1.33(1.09-1.63)] and any tobacco use [AOR: 1.27 (1.05-1.52)]. The odds of smoking tobacco was 83% higher among the most affluent group [AOR: 1.83 (1.26-2.65)] whereas the chances of smokeless tobacco use was 38% higher among the most deprived [AOR: 1.38 (1.01-1.87)].

Table-3 Multivariable association between various forms of tobacco with socio-demographic characteristics.

**Socio-demographic Characteristics**	**Smoking**	**Smokeless Tobacco**	**Any Tobacco**
	**(AOR, 95% CI)**	**(AOR, 95% CI)**	**(AOR, 95% CI)**
Age			
45 to 59 years	Reference	1.01 (0.83-1.22)	Reference
60 to 74 years	1.10 (0.88-1.37)	Reference	1.09 (0.91-1.32)
≥75 years	1.46 (0.99-2.16)	1.65 (1.16-2.36)	1.74 (1.24-2.43)
Gender			
Male	10.04 (7.83-12.87)	1.43 (1.17-1.73)	4.04 (3.32-4.91)
Female	Reference	Reference	Reference
Residence			
Rural	1.42 (0.98-2.07)	1.0 (0.75-1.34)	1.02 (0.77-1.36)
Urban	Reference	Reference	Reference
Education			
No formal education	2.35 (1.64-3.37)	1.47 (0.95-2.28)	3.10 (1.97-4.89)
Up to standard VII	1.96 (1.37-2.82)	1.81 (1.16-2.82)	3.00 (1.90-4.75)
Standard VIII-X	Reference	1.59 (0.99-2.55)	1.72 (1.07-2.77)
Higher secondary & above	0.81(0.48-1.40)	Reference	Reference

Occupation			
Currently working	0.84 (0.65-1.09)	1.08 (0.87-1.35)	1.06 (0.86-1.29)
Currently not working	Reference	Reference	Reference
Life-partner			
Living with partner	1.21 (0.93-1.57)	Reference	0.87(0.71-1.08)
Living without partner	Reference	1.24(1.00-1.53)	Reference
Region			
North	1.91 (1.16-3.15)	Reference	Reference
Central	2.75 (1.97-3.84)	2.42 (1.50-3.91)	2.04 (1.38-3.01)
East	1.17 (0.82-1.66)	6.21 (3.91-9.88)	2.53 (1.70-3.75)
North East	2.16 (1.58-2.95)	3.25 (2.09-5.06)	1.95 (1.36-2.79)
West	Reference	4.01 (2.49-6.44)	1.85 (1.25-2.76)
South	2.31 (1.54-3.46)	1.40 (0.81-2.41)	1.35 (0.87-2.09)
Physical Activity			
Active	0.97(0.77-1.24)	1.33(1.09-1.63)	1.27 (1.05-1.52)
Not Active	Reference	Reference	Reference
Alcohol			
Yes	2.09 (1.69-2.57)	3.05 (2.54-3.66)	4.05 (3.38-4.86)
No	Reference	Reference	Reference
Asset Quintile			
Most deprived	0.96(0.73-1.26)	1.38 (1.01-1.87)	0.96(0.71-1.29)
2	Reference	1.11 (0.81-1.52)	0.86(0.64-1.17)
3	1.74(1.28-2.38)	1.08 (0.78-1.50)	1.10(0.81-1.50)
4	1.73(1.22-2.45)	0.98(0.66-1.45)	0.96(0.66-1.37)
Most affluent	1.83(1.26-2.65)	Reference	Reference

DISCUSSION

This study reported the prevalence and determinants of tobacco use among older adults from indigenous communities of India using nationally representative data. Overall, 46 percent of people use tobacco, with 19 percent of smokers and approximately 32 percent of SLT users. Male, no formal education, central region, alcohol, and most affluent group were all related with smoking, whereas participants over 75 years old, male, eastern area, and alcohol were all

associated with SLT usage. Any tobacco use was associated with males, no formal education, eastern region, and alcohol.

The overall prevalence of tobacco observed in our study is consistent with the findings of a study from Madhya Pradesh which found tobacco was used by 48.1% of urban tribal people aged 40-65 years.(21) Here, it is worth noting that the prevalence of tobacco use among tribal is much higher than that of the general population (28.6%) in India as reported by GATS-2.(5) Another study conducted among the Gond tribe in central India observed the prevalence of smoking to be around 22% which is in harmony with the findings of our study.(22) In contrast with the findings of our SLT prevalence, a study conducted among indigenous communities of Kerala observed the prevalence of SLT use to be around 92%.(14) A probable reason for higher SLT use among tribes could be the socio-cultural acceptance as a part of custom and perceived health benefits of SLT use as compared to smoking. Nonetheless, almost half of our participants consumed nicotine which may lead to deleterious effects on the oral cavity, respiratory system, and cardiovascular system.(23)

We identified older age to be an important predictor of tobacco use among tribal communities which is consistent with findings of a study among nationally representative population-based on GATS and GYTS that reported tobacco dependency was highest among the individuals aged ≥45 years.(24) In our analysis, male participants were more likely to use tobacco than their female counterparts which was similar to the result of a study conducted among various tribes of Kerala.(25) However, a study conducted among the general population suggested smoking to be more prevalent among males while SLT was common among females which makes the determinants of tobacco use different among tribes.(26) Further, we found an association between tobacco use and lesser educational attainment. Smoking tobacco was more common among those who did not have formal education, whereas participants who completed primary education were more frequent users of the smokeless form. These findings are similar to the

results of a study conducted among urban tribal people in Madhya Pradesh which reported maximum tobacco consumption among participants with lesser years of schooling.(21)

A higher risk of smoking was identified among participants from affluent tribal groups. However, a review suggested smoking to be higher in medium wealth quintiles group of the general population.(13) Additionally, it also reported SLT use in India was more among the poorest class.(13) We observed tobacco use was associated with the simultaneous use of alcohol among our study participants. A recent meta-analysis reported that the synergistic consumption of any form of tobacco with alcohol is significantly associated with higher risk of oral squamous cell carcinoma.(27) We observed higher odds of smoking among the central region while the risk of SLT use was more among the eastern region. The regional differences emphasize further strengthening tobacco control programs and policies with a focus on the eastern, central as well as southern tribal populations.

Several existing tobacco control programs and policies such as National Tobacco Control Programme (NTCP) and The Cigarettes and Other Tobacco Products (Prohibition of Advertisement and Regulation of Trade and Commerce, Production, Supply and Distribution) Act, COTPA, 2003 have been effective in reducing the burden of tobacco in India.(28) Still, a wide gap exists between the burden of tobacco consumption among tribal and general population. Our study reflects the prevalence of tribal tobacco use is higher than the average national use which calls for an urgent action in the tribal dominated regions.(5) The existing programs should be tailored in such a way that it is culturally acceptable and linguistically understandable among these groups which can help in creating a positive effect.

Implications

Our study suggests that the prevalence of tobacco use was high among those who had no formal education. So, culturally targeted messages are more effective way to testify positive effect

towards individual health status and control tobacco among indigenous populations.(29) Culturally appropriate and acceptable behavioural change communication (BCC) is required for this group. Folk dances, puppet shows and other such traditions can be employed for better acceptability and interest. Socio-cultural and religious events of particular tribal groups can be used as a platform for communicating anti-tobacco messages. Anti-tobacco campaigns should use messages in local language pertaining to the particular tribal group for their better understanding and arousing interest in the subject matter. In addition, the tribal youths who are literate, local and share a common dialect can actively participate in propagation of tobacco related hazards and associated health risks. Widespread sensitization, attempts to resolve the gap regarding tribal focussed plans as well as leveraging of government programs in context to tribal health may settle the tobacco problem. Although, tobacco consumption is high irrespective of gender, still males are at a higher risk of tobacco use making them the priority target group. Eastern region needs a more strengthened programme owing to the higher burden.

Strength and Limitations

LASI provides a large and nationally representative dataset and also an adequate sample size, providing valuable data on consumption of various forms of tobacco such as smoking, smokeless and any tobacco use and established a strong association between prevalence of tobacco and various sociodemographic factors. We considered the self-reported cases of various forms of tobacco use which may lead to underestimation (misclassification bias) of the actual burden of tobacco.

CONCLUSION

This study highlights a high burden of tobacco use along with its social determinants which can help in tailoring the tobacco control programme among tribal in India. Tobacco control policies should target male, rural residents, individuals with no formal education and alcoholics. Regional variations should be managed by adopting good practices of regions with

higher tobacco control. Future studies should explore the behavioural and social linkages of tobacco use in this group.

Declaration

All methods were carried out following relevant guidelines and regulations.

Ethics approval and consent to participate

The present study utilizes de-identified data from a secondary source. The data has been archived in the public repository of LASI held at IIPS. Access to the data requires registration which is granted specifically for legitimate research purposes. LASI received mandatory ethical approval from the Indian Council of Medical Research and Institutional Review Board (IRB) held at IIPS, Mumbai. Written consent forms were administered at household and individual levels, following the Human Subject Protection. LASI data is archived in a public repository; therefore, there is no need for additional ethical approval to conduct the present study.

Consent for publication

Not applicable

Availability of data and materials

The dataset analysed during the current study is available in the LASI data repository held at ICT, IIPS [https://g2aging.org/?section=overviews&study=lasi].

Conflict of interests

The authors declare that they have no competing interests.

Funding

No funds were received to conduct the present research.

Authors' contributions

Conceptualisation: Srikanta Kanungo, Shishirendu Ghosal, Abhinav Sinha and Sanghamitra Pati. Data curation: Sanghamitra Pati. Formal analysis: Jogesh Murmu, Ritik Agarwal, Sayantani Manna and Sweta Pattanaik. Methodology: Srikanta Kanungo, Abhinav Sinha, Shishirendu Ghosal, Ardhendu Sekhar Acharya and Sanghamitra Pati. Software: Srikanta Kanungo and Sanghamitra Pati. Supervision: Srikanta Kanungo and Sanghamitra Pati. Visualisation: Jogesh Murmu, Ritik Agarwal, Sayantani Manna, Sweta Pattanaik and Shishirendu Ghosal. Writing - original draft: Jogesh Murmu, Ritik Agarwal, Sayantani Manna, Sweta Pattanaik, Shishirendu Ghosal and Abhinav Sinha. Writing - review & editing: Srikanta Kanungo, Ardhendu Sekhar Acharya and Sanghamitra Pati.

Acknowledgments

The authors are grateful to the Longitudinal Ageing Study in India (LASI), for assembling and publishing accurate, nationally representative data on a range of health, biomarkers, and healthcare utilization indicators for the population in the age range of 45 years and older. The authors are also grateful to LASI's project partners, the International Institute for Population Sciences (IIPS), Harvard T. H. Chan School of Public Health (HSPH), and the University of Southern California (USC).

REFERENCE

1. Tobacco [Internet]. [cited 2021 Nov 20]. Available from: https://www.who.int/news-room/fact-sheets/detail/tobacco
2. Mohan P, Lando HA, Panneer S. Assessment of Tobacco Consumption and Control in India. Integrative Medicine Insights. 2018 Mar 2;9.
3. Mishra GA, Pimple SA, Shastri SS. An overview of the tobacco problem in India. Indian Journal of Medical and Paediatric Oncology. 2012;33(3):139–45.
4. Gupta PC. Report on Tobacco Control in India Edited by.
5. Adult G, Survey T. Gats 2. 2016;6–9.
6. Ministry of Health & Family Welfare Government of India. Global Adult Tobacco Surey 2016-2017 [Internet]. Vol. 1, International Institute for Population Sciences. 2017. 1–314 p. Available from: https://mohfw.gov.in/sites/default/files/GlobaltobacoJune2018.pdf

7. Kamal N, Faheem M, Fazlullah K, Haji B, Fatima IH, Mohammad A. Smokeless tobacco (paan and gutkha) consumption, prevalence, and contribution to oral cancer. Epidemiology and Health. 2013;(1):1–11.

8. Tobacco Free Kids. Tobacco burden facts. 2013;23(August):2013. Available from: http://global.tobaccofreekids.org/files/pdfs/en/Indonesia_tob_burden_en.pdf

9. Islands N, Haveli N. Table 2 : Literacy Rates among STs and ALL.

10. Issue S. Regional Medical Research Centre for Tribals World Health Organization Collaborating Centre for the Health of Indigenous Populations Example : 2014;20(January). Available from: www.icmje.org

11. Ministry of Tribal Affairs. Tribal Health Expert Committee Report Executive Summary [Internet]. Ministry of Tribal Affairs. 2018. p. 54. Available from: http://nhm.gov.in/nhm_components/tribal_report/Executive_Summary.pdf

12. John RM. Price elasticity estimates for tobacco products in India. Health Policy and Planning. 2008 May;23(3):200–9.

13. Thakur JS, Paika R. Determinants of smokeless tobacco use in india. Vol. 148, Indian Journal of Medical Research, Supplement. Indian Council of Medical Research; 2018. p. 41–5.

14. C J, J J, S V, F T, CV DK, R V, et al. Prevalence and Dependency of Tobacco Use in an Indigenous Population of Kerala, India. Journal of Oral Hygiene & Health 2016 4:1 [Internet]. 2016 Feb 7 [cited 2021 Dec 8];4(1):1–4. Available from: https://www.omicsonline.org/open-access/prevalence-and-dependency-of-tobacco-use-in-an-indigenous-populationof-kerala-india-2332-0702-1000198.php?aid=69141

15. Chellappa L, Leelavathi L, Indiran M, Rathinavelu P. Prevalence and dependency of tobacco use among tribal gypsies in Thoothukudi district - A cross sectional study. Journal of Family Medicine and Primary Care. 2021;10(2):738.

16. National_Fact_Sheet_of_fourth_round_of_Global_Youth_Tobacco_Survey_GYTS-4.pdf.

17. Grover S, Anand T, Kishore J, Tripathy JP, Sinha DN. Tobacco Use Among the Youth in India: Evidence From Global Adult Tobacco Survey-2 (2016-2017). Tobacco Use Insights. 2020;13:1179173X2092739.

18. LASI. Longitudinal Ageing Study in India (LASI) INDIA EXECUTIVE SUMMARY NATIONAL PROGRAMME FOR HEALTH CARE OF ELDERLY & INTERNATIONAL INSTITUTE FOR POPULATION SCIENCES MINISTRY OF HEALTH & FAMILY WELFARE GOVERNMENT OF INDIA. 2017;

19. Longitudinal Ageing Study in India (LASI) INDIA REPORT NATIONAL PROGRAMME FOR HEALTH CARE OF ELDERLY & INTERNATIONAL INSTITUTE FOR POPULATION SCIENCES MINISTRY OF HEALTH & FAMILY WELFARE GOVERNMENT OF INDIA.

20. STROBE - Strengthening the reporting of observational studies in epidemiology [Internet]. [cited 2022 Mar 25]. Available from: https://www.strobe-statement.org/

21. Gupta DVK. Prevalence of Tobacco Consumption among Urban Tribals of Mandla District (M.P). Journal of Medical Science And clinical Research [Internet]. 2018 Feb 12;6(2). Available from: http://jmscr.igmpublication.org/v6-i2/67%20jmscr.pdf

22. Rao VG, Bhat J, Yadav R, Muniyandi M, Bhondeley MK, Wares DF. Smoking and alcohol consumption: Risk factors for pulmonary tuberculosis among the tribal community in central India. Indian Journal of Tuberculosis. 2017 Jan 1;64(1):40–3.

23. Bartal M. Heath effects of tobacco use and exposure [Internet]. Available from: https://www.researchgate.net/publication/11389729

24. Chhabra A, Hussain S, Rashid S. Recent trends of tobacco use in India. Vol. 29, Journal of Public Health (Germany). Springer Science and Business Media Deutschland GmbH; 2021. p. 27–36.

25. KC D, Jose M, Prabhu V. Prevalence and Type of Tobacco Habits and Tobacco Related Oral Lesions among Wayanad Tribes, Kerala, India. Indian Journal of Public Health Research & Development [Internet]. 2013 Apr;4(2):63. Available from: http://www.indianjournals.com/ijor.aspx?target=ijor:ijphrd&volume=4&issue=2&article=015

26. Kankaria A, Sahoo SS, Verma M. Awareness regarding the adverse effect of tobacco among adults in India: Findings from secondary data analysis of Global Adult Tobacco Survey. BMJ Open. 2021 Jun 28;11(6).

27. Mello FW, Melo G, Pasetto JJ, Silva CAB, Warnakulasuriya S, Rivero ERC. The synergistic effect of tobacco and alcohol consumption on oral squamous cell carcinoma: a systematic review and meta-analysis. Vol. 23, Clinical Oral Investigations. Springer Verlag; 2019. p. 2849–59.

28. Ghosal S, Sinha A, Kanungo S, Pati S. Declining trends in smokeless tobacco use among Indian women: findings from global adult tobacco survey I and II. BMC Public Health. 2021 Dec 1;21(1).

29. Gould GS, McEwen A, Watters T, Clough AR, van der Zwan R. Should anti-tobacco media messages be culturally targeted for Indigenous populations? A systematic review and narrative synthesis. Vol. 22, Tobacco Control. 2013.

Selection of study population

Click here to access/download;Figure;Fig-1.tiff

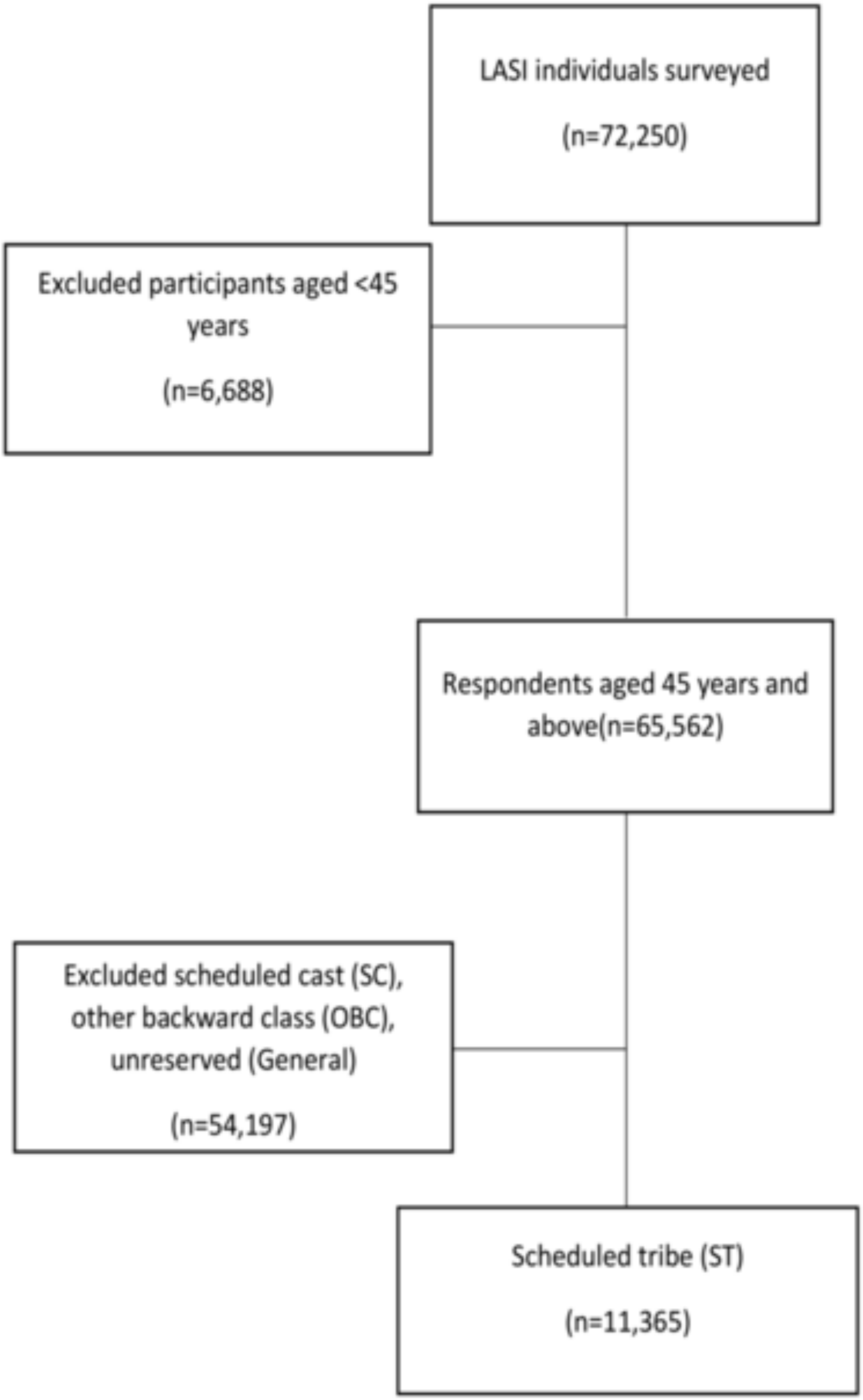

Type of tobacco consumption across gender among the tribal populations

Click here to access/download;Figure;Fig-2.tif

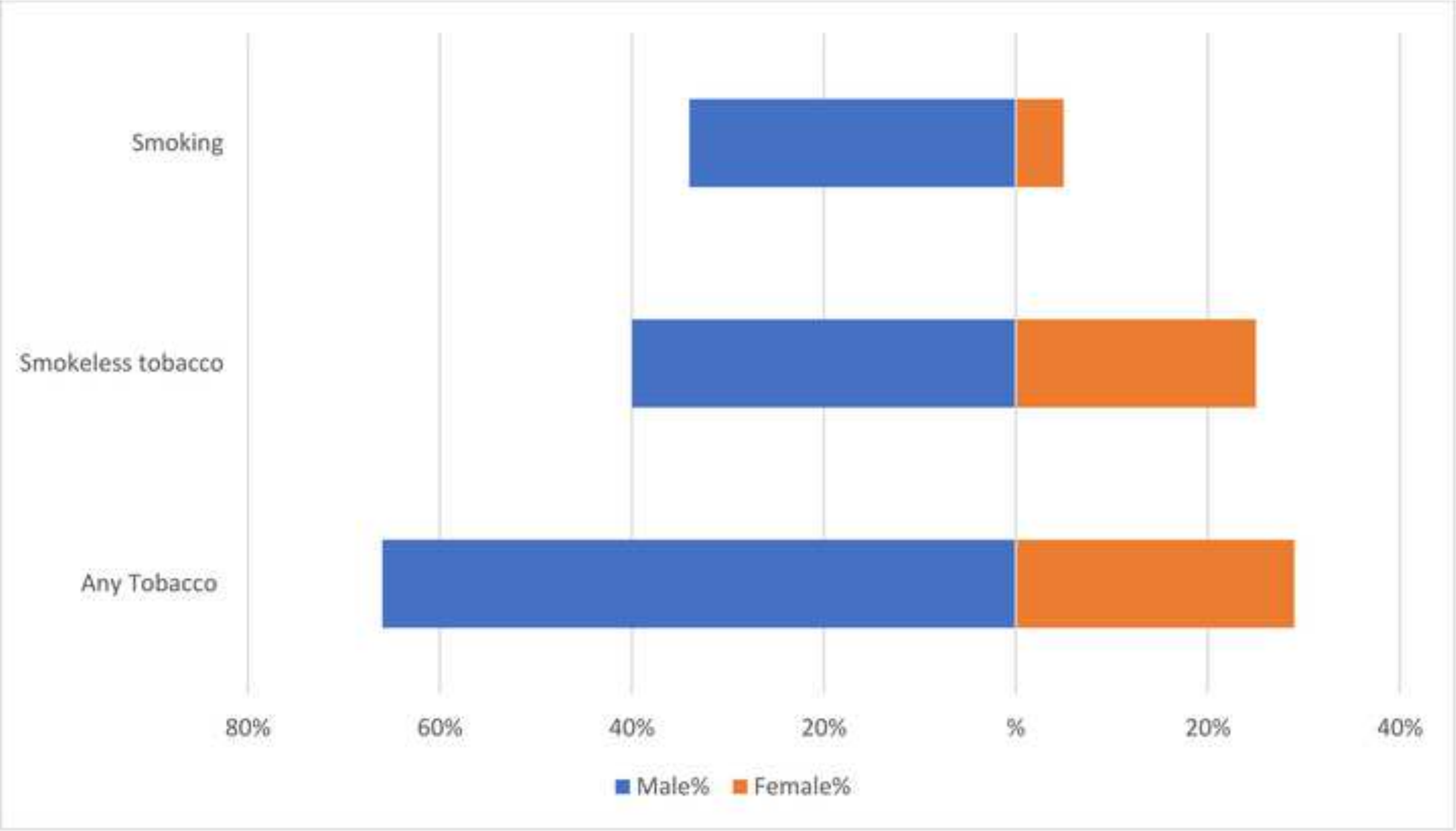

State wise prevalence of smoking tobacco among tribal population in India

Click here to access/download;Figure;Fig-3a SMOKING.tiff

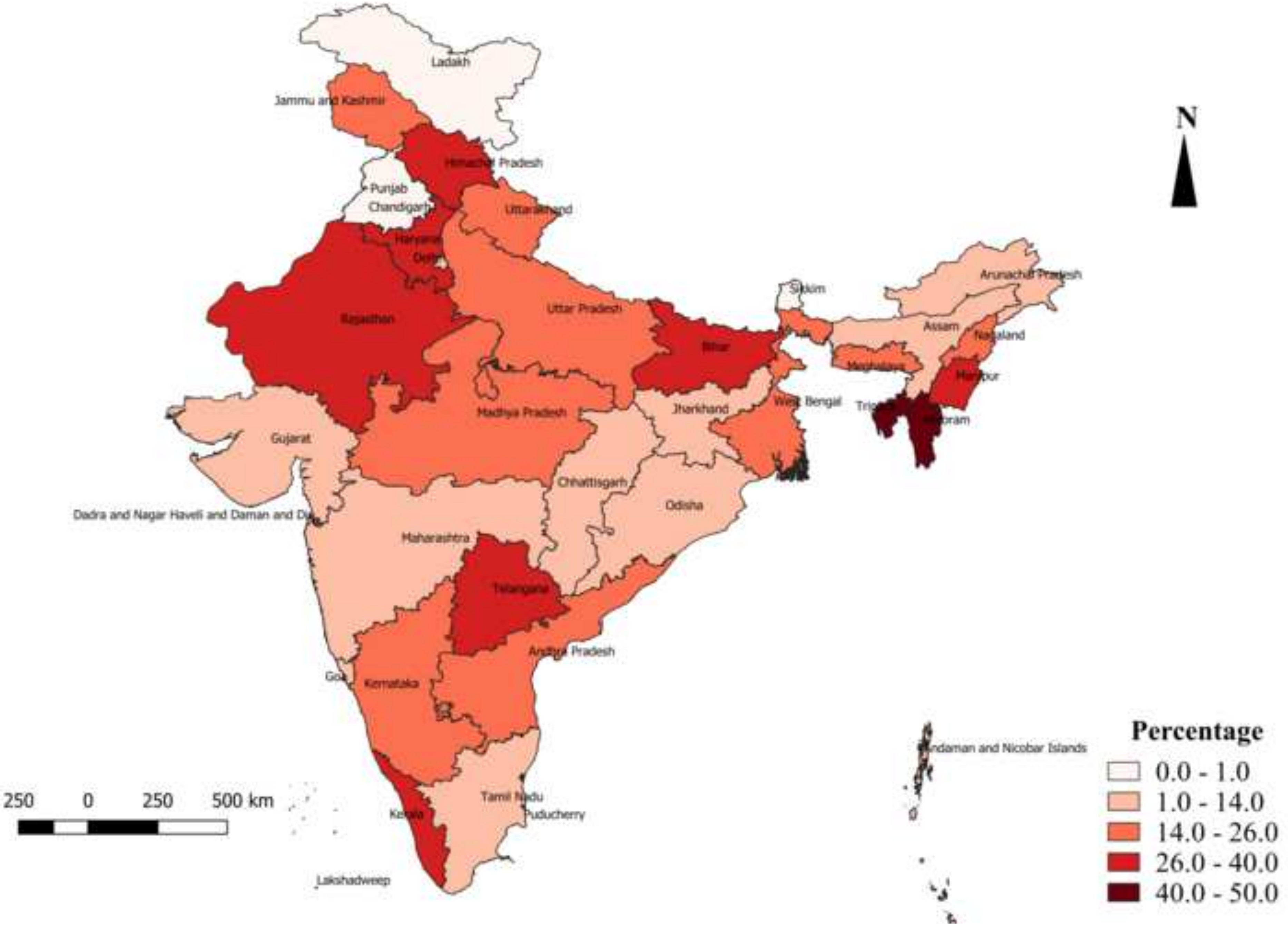

: State wise prevalence of smokeless tobacco among tribal population in India

Click here to access/download;Figure;Fig-3b SMOKELESS.tiff

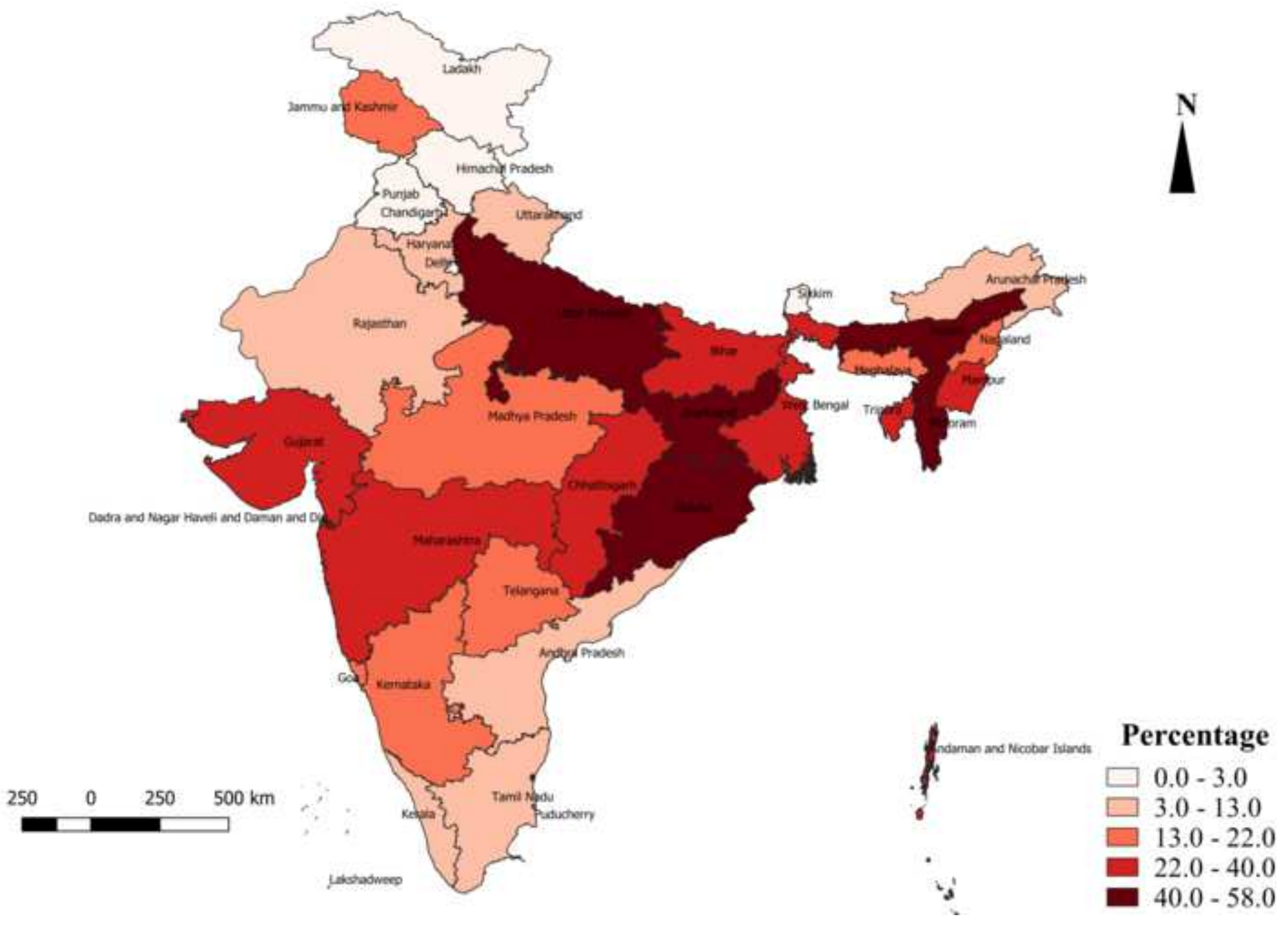

: State wise prevalence of any tobacco among tribal population in India

Click here to access/download;Figure;Fig-3c ANY TOBACCO.tiff

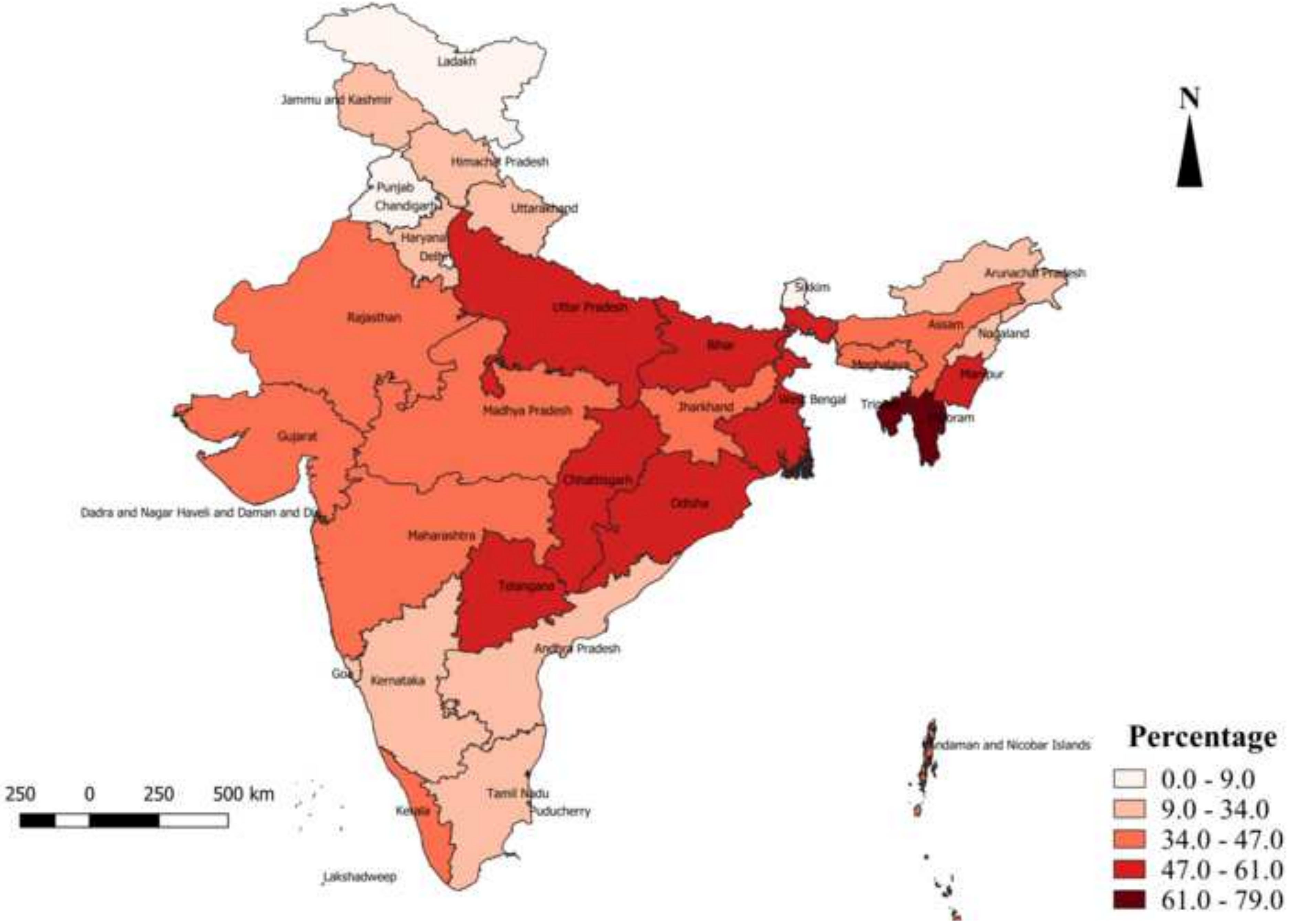

Click here to access/download
Supporting Information
S1-Table strobe checklist.doc

State wise prevalence table

Click here to access/download
Supporting Information
S2-Table Prevalence table.docx
