# Supplementary material for: Social determinants of tobacco use among tribal communities in India: Evidence from the first wave of Longitudinal Ageing Study in India
Source: PLoS One. 2023 Mar 2;18(3):e0282487. doi: 10.1371/journal.pone.0282487 (PMC9980830; doi:10.1371/journal.pone.0282487)
Supplement: Attachment — Submitted filename: Response to Reviewers.docx [file pone.0282487.s005.docx]

**Point-by-Point Response Reviewers’ Comments**

We would like to thank the Editor and reviewers for their thoughtful comments and efforts towards improving our manuscript. The suggestions offered have been extremely helpful in revising the manuscript. We have incorporated all the changes suggested. Please find the response to all the comments highlighted in a point-by-point basis. We hope the revised manuscript is appropriate for publication in the journal. But we are open to further revisions if required.

**Reviewer #1**: There are few things to be corrected within the already written paper, which were also highlighted in supporting reviewed file.
The authors must go through following points:
**1. Go through some already published plos one paper's and make proper citation.**

*Authors’ reply*- Thank you for your suggestions we have gone through the literature and changed our citations accordingly.
**2. For some words use synonyms or different words.**

*Authors’ reply-* We have tried to improve the entire manuscript by editing it and we have added synonyms also as suggested by the reviewer.
**3. Mention why you have categorized age as 14 years difference, the answer may be supported by previous literature.**

*Authors’ reply*- We have categorized age in line with the previous literatures which have now been cited in the manuscript.
**4. If the authors have taken "living arrangement variable" for 'living with partner' and 'living without partner', then how the authors manage 'living alone category'. Also, in independent variable/covariates section, mention the name of "variable taken from LASI", and how you have recoded the variables for your study**.

*Authors’ reply*- Initially we had taken dm005 (current marital status) as our partner status variable. But as suggested by the reviewer, we have taken the new variable from the LASI dataset which is the “living arrangement variable” to include the category of living alone.
**5. Go through some already published plos one paper's and make tables properly. Also, use Inactive instead of not active, its sound better in research article**.

*Authors’ reply*- As per the suggestions of the reviewer we have changed the tables according to plos-one guidelines and included standard fonts and standard size. Additionally, for physical activity we have changed not active to inactive.

**6. In table 2, authors have make a haphazard table. Please try to take the same category in reference among all parameters. Its better to re-do table 2, specially for variables like- age, education, life-partner, religion, and asset quintile. If not agreed for re-doing then give valid reason for keeping such vigorous category referencing in the methodology section.**

*Authors’ reply*- Previously we had taken different reference groups as mentioned by the reviewer. However, as per the suggestions we have redone the table-2 (univariate regression analysis) and we have kept uniform reference groups of all the models.

**7. Asset quintile category should be named in a similar fashion, keep same type of naming for all five categories. Don't use 2,3,4 suddenly in between most deprived and most affluent. Authors may use (extremely, less, medium, high, very high) type of categorization**.

*Authors’ reply*- Thank you for the suggestion. We have changed the names of five categories of asset quintile to poorest, poorer, middle, richer, and richest.

**8. Do not use 'our participants'. Use correct English. Authors may use 'study participants'.**
*Authors’ reply*- Thank you for pointing this out. We have now revised it with study participants in our manuscript.

**There are few things that are required to do for this paper,
1. Most of the literature cited were taken from less prevalent states. (Example- Madhya Pradesh, & Kerala). But north-eastern are highly tobacco prevalent states. Please go through some papers on north-eastern states, there are plenty of works available for north eastern states.**

*Authors’ reply*- We agree with the reviewer’s suggestions. However, most of the literature from north eastern region mainly focussed either on general population or on tribal adolescents with almost no study in context to older tribal adults which makes our study incomparable with them. This has forced us to not cite those studies in our manuscript and also this is the novelty of our study that it focuses on an age group in which less attention has been paid till now as most studies focus on young adults.

**2. In conclusion writing a line like (Regional variations should be managed by adopting good practices of regions with higher tobacco control) is not justified. Authors must name the states where government intervention is required.**

*Authors’ reply*- Thank you for your suggestions. This is an important point that we have missed and it is worth noting the name of the states where the burden of tobacco use is high and we have addressed this point in the conclusion under the regional variations paragraph.

**3. Authors have perfectly done a regional analysis. But naming the states within that region is necessary. For example, there is high prevalence of tobacco in north eastern states, and Assam government banned tobacco products on 22 November 2019. Then Tripura government can take similar steps, is a conclusion. Authors may write conclusion section in such a way.**

*Authors’ reply*- The reviewer has raised a very valid point and we have now mentioned the names of the states. Additionally, in the discussion section we have discussed on the similar lines as suggested by the reviewer.

**4. Last but not least, authors should concentrate more on high tribal population states like Madhya Pradesh, Orissa, Maharashtra, Rajasthan, & Chhattisgarh. Along with a higher percentage of Tribal population states like Arunachal Pradesh, Nagaland, Mizoram, Meghalaya, & Manipur**.

*Authors’ reply*- Thank you for the suggestion. We have now focussed on high tribal populated states and mentioned it in the discussion section.

We again thank the reviewer for their valuable time and inputs.

**Reviewer #2**: **In this manuscript, authors have made an attempt to estimate the prevalence of tobacco use among tribal population aged 45 years or above in India based on secondary data-LASI. Efforts shown by the authors are good but there some major concerns in this current version of the manuscript listed below**:

**1. The literature search appears less robust and authors have missed on many recent publications. The high prevalence of tobacco use among this socially vulnerable group has been already established in the last literature and information provided here is not adding anything new to the existing knowledge. Therefore, rationale to conduct this study on tobacco use among aged tribal population needs a stronger rationale**.

*Authors’ reply*- We appreciate the observations of reviewer. However, we affirm that the available literature among the tribal focuses mainly on younger age groups with a scarcity of data among older adult. We generated evidence using a nationally representative data of older tribal adults which is novel and a strength of this study.

**2. The citations need to be updated and should follow a specific format as per the journal guideline. There are also some repetitions or in a format difficult to understand say for example 18. 19**

*Authors’ reply*- We have gone through the guidelines of plos one for citation of articles and have modified the references accordingly.

**3. In the methodology section, the analysis part seems incomplete. Even for regression analysis, what covariates were adjusted were not clear. The association between alcohol consumption and tobacco use might be misleading unless controlling covariates are clearly mentioned. It is also not clear how did they adjust for sample weights. The presentations on the table appear much cluttered and hard to comprehend.**

*Authors’ reply*- For multivariable regression analysis we have adjusted for all the socio-demographic variables. WE have now mentioned this in the statistical analysis portion of the methodology. Sampling weights have been utilized in entire analysis to compensate for the complex survey design which has now been mentioned in the manuscript for better understanding of the readers.

**4. Apart from misclassification error, there are possibilities of other potential sources of biases which the authors didn't mention and also how did authors try to adjust for such biases is also not mentioned**

*Authors’ reply*- Thank you for your suggestion. We have now addressed other potential biases also.

**5. The overall writing style and presentations need to be more comprehensive**

*Authors’ reply*- As per your suggestion we have now modified the entire manuscript to check for grammar, punctuations in order to enhance writing and presentations style.

We again thank the reviewer for their valuable time and inputs.

**Reviewer #3**: **The paper is trying to address an important question on tobacco use behaviour of the tribal population in India and understand their determinants within this group. However, I do have some major comments to improve this paper.**

**1. This paper is based on LASI data which is nationally and state representative. However, since this study is conducted on a specific set of population, any estimation for ST population at the state level may not be appropriate, as the total sample of ST population in this data is 11,365 tribal individuals aged ≥ 45 years. Segregating this data at the state level will lead to very few cases in smaller states. Therefore, I would recommend Authors to remove Figure 3 completely from this paper. If they want, they can provide a table of prevalence of tobacco use in bigger states only in the form of a table in place of Figure 3.**

*Authors’ reply*- As per your suggestions, we have removed the figure-3 (State-wise prevalence of various forms of tobacco among tribals in India). Instead of that, we have added the state-wise prevalence of various forms of tobacco in the supplementary file and also added the figure of top 4 tribal populated states according to census-2011 population.

**2. I also fail to understand the reason for taking difference reference group each time for wealth quintile for tobacco, Smokeless and smoking outcome. Similarly, west region was used as reference for smoking and North for Smokeless and tobacco use. Can the Authors provide their reason for changing references in these models for the same socio demographic characteristics, or they should make these reference consistent and re do the logistic regression analysis, otherwise it may be difficult to compare the results for different form of tobacco use and their association with these characteristics**.

*Authors’ reply*- Thank you for this valuable suggestion. We have changed the reference groups of the variables where the reference seems to be inconsistent like age, asset quintile, education age, region, living arrangement variables. And we have redone table-2 and kept the reference group same as suggested by the reviewer.

**3. Are there any evidence which suggest that physical activity leads to higher/lower tobacco use. I did find literature the other way around. Authors should provide some appropriate references to substantiate their choice of physical activity as one of the variables.
Jeon, H. G., Kim, G., Jeong, H. S., & So, W. Y. (2021, February). Association between Cigarette Smoking and Physical Fitness Level of Korean Adults and the Elderly. In Healthcare (Vol. 9, No. 2, p. 185). MDPI.
Conway, T. L., & Cronan, T. A. (1992). Smoking, exercise, and physical fitness. Preventive medicine, 21(6), 723-734.**

*Authors’ reply*- The choice of physical activity as one of the variables as most of the tribals are involved in vigorous activities and it was mentioned in detail in the methodology section. However, the suggested literature have been used to compare our findings in the discussion section.

**4. GATS-2 has collected information about tobacco consumption and provides caste-wise data as well. Why the authors have chosen LASI data for this specific study needs to be mentioned clearly. For example, GATS collected selective socioeconomic characteristics data while LASI provides a detailed information on this front.**

*Authors’ reply*- We have given justification for selection of LASI data for our analysis.

**5. Authors can also provide some comparison of GATS-2 and LASI tobacco use data for ST population in the discussion.**

*Authors’ reply*- We have addressed the comparison in the discussion section and compared the GATS-2 and LASI tobacco use data for the scheduled tribes population.

We again thank the reviewer for their valuable time and inputs.
