# Supplementary material for: Social determinants of tobacco use among tribal communities in India: Evidence from the first wave of Longitudinal Ageing Study in India
Source: PLoS One. 2023 Mar 2;18(3):e0282487. doi: 10.1371/journal.pone.0282487 (PMC9980830; doi:10.1371/journal.pone.0282487)
Supplement: Attachment — Submitted filename: Response to Reviewers3.docx [file pone.0282487.s006.docx]

**Point-by-Point Response Reviewers’ Comments**

We would like to thank the Editor and reviewers for their thoughtful comments and efforts towards improving our manuscript. The suggestions offered have been extremely helpful in revising the manuscript. We have incorporated all the changes suggested. Please find the response to all the comments highlighted in a point-by-point basis. We hope the revised manuscript is appropriate for publication in the journal. But we are open to further revisions if required.

**Reviewer #4**: This present study deals with an important aspect of social determinants of tobacco use among tribals in India. The paper is revised by the authors after the first round of review. However, I have the following comments on the paper:

**1.** In the title, “Social determinants of tobacco use among tribal communities in India: evidence on ethnicity and tobacco use from LASI, wave-1”, the term like ‘tobacco use’ is repetitive. Again, the term tribal has already been mentioned in the title, the term ‘ethnicity’ may not be required. The title may be simplified to ‘Social determinants of tobacco use among tribal communities in India: evidence from the first wave of LASI (full form??)’.

*Authors’ reply*- Thank you for your suggestion. We have changed the title of the study as suggested.

**2.** Why were smokers and users of tobacco in dual for clubbed into smokers? They are one more dangerous category. The study might have a greater weightage if the severity of the tobacco consumption is considered like only smokeless/other form users, only smokers and both smokers and non-smokers.

*Authors’ reply-* when we created the binary variable "smokers" as an outcome variable, both only smokers and dual users were merged; the sub-groups under smokers were smokers and non-smokers (includes smokeless tobacco users but don't use smoking tobacco and those who don't use any kind of tobacco products). Similarly, when we created another binary variable as "smokeless tobacco users", the subgroups under this variable were SLT users (only SLT users + dual users, as they used SLT products also) and non-SLT users (included those who didn't use tobacco of any kind & those who were consumer of smoking tobacco only). Any tobacco group included all kind of tobacco users (only smokers+ only SLT users + dual users) irrespective of type of products they were using. We conducted three separate logistic regression model for smokers, smokeless tobacco users and any tobacco users to avoid collinearity.

**4.** The sampling weights given in LASI are meant for the general population and may not be applicable to the tribal population.

*Authors’ reply*- We agree with the reviewer. However, studies such as “Puri P, Pati S. Exploring the Linkages Between Non-Communicable Disease Multimorbidity, Health Care Utilization and Expenditure Among Aboriginal Older Adult Population in India. International Journal of Public Health. 2022:5.” have utilized survey weights in analysing LASI data for tribal population. We feel weighted analysis can better present the exact scenario; hence we have used weighted analysis. Nonetheless, following reviewers’ suggestion in methods section we have mentioned this as one of the limitations of the study.

**5.** P10 L212: ‘For this analysis, 11,365 scheduled tribes aged 45 years and above were included.’ I think you mean scheduled tribe participants here?

*Authors’ reply*- We have added the word “participants” as suggested by the reviewer.

**6.**  It should be clearly mentioned in the methodology that the wealth quintiles have been generated from the general population, not from the tribal population.

*Authors’ reply*- As suggested by the reviewer, we have mentioned that the wealth quintiles are based on monthly per capita expenditure of the household from the general population, not from the tribal population.

**7.** Table 2 headings are given as n (%) but data is reflected in n, % (CI). The variable names may be aligned to left (Age, Gender etc). Why are one reference category at the top and the other at the bottom?

*Authors’ reply*- As per the suggestions by the reviewer we have changed the headings and subheadings in table-2 and wherever it is required in our study. We have placed all the reference groups in bottom but in age category the 1^st^ category (i.e., 45-59 years) was taken as reference, hence it remained at the top of the table.

**8.**Why do tribal groups from eastern regions have the highest tobacco consumption? This may be reflected from the existing literature. Does it reflect socio-cultural upbringing of any major tribal group/s?

*Authors’ reply*- We have now added this in the discussion section.

**9.**  Proper referencing should be done for some of the references like “National_Fact_Sheet_of_fourth_round_of_Global_Youth_Tobacco_Survey_GYTS- 4.pdf.”

*Authors’ reply*- Thank you for pointing this out. All references have been revised and re-checked.

10. Please correct the spelling of state names like Maharashtra and Rajasthan in Figure-3

*Authors’ reply*- As per the reviewer’s suggestion all the spelling has been corrected in the figure-3

11. Manuscripts may be carefully edited for scientific language and grammatical errors.

*Authors’ reply*- Manuscript has been checked for grammatical error and corrected accordingly.

We again thank the reviewer for their valuable time and inputs.
