# Supplementary material for: Social determinants of tobacco use among tribal communities in India: Evidence from the first wave of Longitudinal Ageing Study in India
Source: PLoS One. 2023 Mar 2;18(3):e0282487. doi: 10.1371/journal.pone.0282487 (PMC9980830; doi:10.1371/journal.pone.0282487)
Supplement: Attachment — Submitted filename: Response to Reviewers.docx [file pone.0282487.s008.docx]

**Response to Reviewers**

We would like to thank the Editor and reviewers for their thoughtful comments and efforts toward improving our manuscript. The suggestions offered have been extremely helpful in revising the manuscript. We have incorporated all the changes suggested. Please find the response to all the comments highlighted on a point-by-point basis. We hope the revised manuscript is appropriate for publication in the journal. But we are open to further revisions if required.

**Academic Editor's Comments:**

**1. The revised manuscript still needs language editing in some of its sections. Please follow some of the editing done in the attached copy of the manuscript (with track change).**

*Author’s Response:* Thank you for your suggestion. We have changed the revised manuscript with language editing as suggested by the editors. In lines 259-260: as suggested by the editor, we have replaced smokeless with SLT and followed with throughout the manuscript.

**2. In line 185-187: Authors have used the sentence “Wealth quintiles (poorest, poorer, middle, richer, richest) were based on the monthly per capita expenditure of the general population.” LASI data does not provide wealth quintile; it is MPCE quintile and they can’t be used interchangeably. So, replace the wealth quintile with the MPCE quintile, wherever used.**

**Authors can avoid the limitation of using the indicator calculated based on the general population by calculating the MPCE quintile for their sample (tribal population) as well. This can be easily done using the expenditure details on food and non-food items including spending on health, education, utilities etc. given in the household dataset.**

*Author’s Response:* Thank you for this important insight. We understand the concern raised by the editor, in this comment. The analysis was based on the individual dataset and there were several households, from where more than one individual was interviewed.

Using the household dataset for calculating MPCE might repeat (if: both husband and wife were included) or not repeat (if two or more siblings from the same household were included) those variables required for principal component analysis, which we wanted to avoid here.

**3. Follow the other comments and suggestions mentioned in the attached edited copy of the manuscript.**

*Author’s Response:* Thank you for your valuable suggestion. We have addressed suggestions made by the editor, particularly in regard to adding references, reframing the sentences, and highlighting changes done.
